# Supplementation with hArg During the Rapid Growth of the Placenta Modulates Final Placental Angiogenesis and Pregnancy Outcomes

**DOI:** 10.3390/nu17223563

**Published:** 2025-11-14

**Authors:** Huijuan Li, Feng Yong, Lixue Liu, Na Ren, Rui Han, Tianrui Zhang, Dongsheng Che

**Affiliations:** Jilin Provincial Science and Technology Innovation Center of Pig industry Technology, Jilin Provincial Key Laboratory of Animal Nutrition and Feed Science, Key Laboratory of Animal Production, Product Quality and Security, Ministry of Education, College of Animal Science and Technology, Jilin Agricultural University, Changchun 130118, China; js9794lyy@163.com (H.L.); yongfeng0485@163.com (F.Y.); llxlns8888@163.com (L.L.); rn_7260@163.com (N.R.); hanrui0409@163.com (R.H.)

**Keywords:** placenta, homoarginine, angiogenesis, pregnancy outcomes

## Abstract

Background: Placental angiogenesis is crucial for ensuring placental function and normal fetal development. It has been demonstrated that elevated plasma levels of homoarginine (hArg), an endogenous amino acid, during pregnancy correlate with enhanced vascular endothelial function. However, the effect of hArg in placental angiogenesis during pregnancy is still ambiguous. This study seeks to evaluate the impact of supplemental hArg during the rapid placental growth phase in early pregnancy, a key moment for placental angiogenesis, on ultimate pregnancy outcomes and placental angiogenesis in rats, as well as its potential processes. Methods: This study utilized thirty 8-week-old female Sprague Dawley rats as pregnant animals, which were randomly allocated to control and treatment groups (15 per group) and administered 20 mg/kg of hArg from embryonic day 0.5 (E0.5) to E13. The dams were euthanized on E21. Results: Maternal hArg dietary supplementation positively influenced pregnancy outcomes, resulting in a significant increase in the number of live-born offspring and total placental weight, alongside elevated maternal circulating reproductive hormone levels. Additionally, the upregulation of the amino acid transporter in the placenta of the treatment group established a basis for hArg accumulation in the placenta, hence promoting hArg-specific augmentation of eNOS-mediated NO production. The treated group simultaneously demonstrated an expanded labyrinthine zone, increased blood sinusoids area, enhanced vascular density, and raised levels of proangiogenic factors. Mechanistically, hArg enhanced the expression of proteins linked with the PI3K/AKT signaling pathway. Conclusions: Supplementation with hArg during the placenta’s rapid growth phase enhances placental angiogenesis, ultimately enhancing pregnancy outcomes. This effect may be attributed to the regulation of the PI3K/AKT signaling pathway.

## 1. Introduction

During the intricate physiological period of pregnancy, a temporary organ forms between the mother and fetus: the placenta. As a key participant in the pregnancy process, the rapid growth and functional maturation of the placenta form the cornerstone for determining maternal and fetal health and pregnancy outcomes [1]. Among them, placental angiogenesis is essential for maintaining placental blood flow. This process ensures the placenta’s proper food exchange between mother and fetus, hormone production, gas exchange, and waste elimination, therefore fulfilling the requirements for fetal growth and development [2]. Angiogenesis is defined as a multistep dynamic biological process that entails the development of new blood vessels from existing ones [3,4]. Placental angiogenesis predominantly transpires in early pregnancy, marked by swift cellular proliferation and differentiation [5,6]. Starks et al. [7] revealed that by embryonic day 9.5 (E9.5) in rats, the placenta is developing a complex vascular network. At E12.5, the early placenta transitions towards maturity, with cellular proliferation activity reaching its peak in the placental labyrinth zone (LZ), resulting in the formation of a dense vascular network [8,9,10]. Consequently, given the critical window of early pregnancy for placental angiogenesis, the relationship between precise regulation of maternal nutrition during this phase and pregnancy outcomes has garnered extensive attention.

As fundamental units of protein synthesis, cellular signaling, and metabolic regulation, the adequate supply and efficient utilization of amino acids are crucial for placental angiogenesis [2]. The homoarginine (hArg) is a homolog of arginine (Arg) [11]. Clinically, hArg has been established as a robust biomarker, with diminished circulating levels correlated with heightened cardiovascular and cerebrovascular risk and mortality [11,12,13,14,15]. Moreover, accumulating evidence indicates that supplementation with hArg at supraphysiological levels yields beneficial effects on cardiovascular and cerebrovascular outcomes. For instance, numerous investigations utilizing chronic heart failure mice models have found that hArg supplementation mitigates left ventricular systolic reserve and hemodynamic dysfunction [16,17]. The hemodynamic parameter-relieving effect of hArg was also corroborated in the study conducted by Faller et al. [18]. Nitz et al. [19] concurrently demonstrated that hArg inhibits the formation of atherosclerotic plaques. Additionally, in murine models of neurological damage, hArg supplementation reduced cerebral infarction volume [11]. Notably, research on pregnancy has revealed a significant positive correlation between hArg and maternal brachial artery diameter and endothelial function, a link absent in non-pregnant women [20]. Evidence indicates that certain vascular advantages of hArg may be facilitated by increasing nitric oxide (NO) availability [12,21]. NO serves as a crucial regulator of angiogenesis, placental development, and embryonic growth [22]. These studies collectively imply numerous advantageous benefits of hArg on vascular function. However, the exploration of hArg’s influence on placental angiogenesis during pregnancy and its subsequent regulation of pregnancy outcomes remains limited.

Our research primarily examines the impact of hArg supplementation during the rapid period of placental growth on placental development, specifically angiogenesis, while also evaluating its potential to optimize pregnancy outcomes. To explore this hypothesis, pregnant rats were administered a dietary supplement of 20 mg/kg hArg from E0.5 to E13, a dosage determined through preliminary dose–response screening and consistent with dosage levels previously shown to be vasoprotective [11,18,19]. The present research employed a multidimensional assessment method to evaluate the impact of hArg on pregnancy outcomes and to clarify its potential mechanisms connected to the placenta. This provides important theoretical support for deepening the understanding of hArg’s potential mechanisms and application value in pregnancy health.

## 2. Materials and Methods

### 2.1. Experimental Animal Design

All animal procedures adhered to the China Council on Animal Care guidelines and were approved by the Animal Care and Use Committee of Jilin Agricultural University (Ethical Approval Code: KT2024078; Approval Date: 8 July 2024).

**Figure 1:**
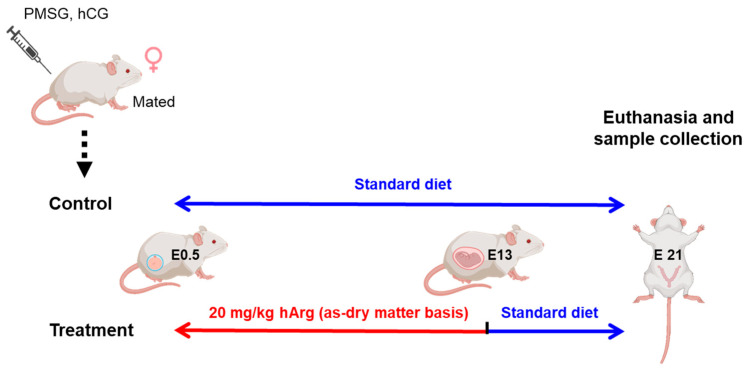
The experimental design and processing timeline. After estrus synchronization and mating, female rats confirmed to be at embryonic day 0.5 (E0.5) were randomly and evenly separated into two groups. The control group was fed a basal diet throughout the pregnancy, while the treatment group received 20 mg/kg hArg during the rapid placental growth stage (E0.5–E13) and then returned to the basal diet. Both groups of pregnant rats were euthanized at E21 to collect samples for subsequent experiment. Created in BioRender. Yong, F. (2025) https://BioRender.com/rjieagf (accessed on 27 September 2025).

Healthy male and female Sprague Dawley rats, each with comparable body weights of 250 ± 10 g, were acquired from Liaoning Changsheng Biotechnology Co., Ltd. (Shenyang, China). All rats were housed under standard environmental conditions, including being specific-pathogen-free, a temperature of 25 °C, and a relative humidity of 50%, while being maintained on a 12 h light-dark cycle. Following a 1–2 week adaptation period, male and female rats were caged together in a 1:2 ratio for a duration of 12 h. Pregnant rats at E0.5 were identified by the presence of vaginal plugs or the detection of a significant quantity of sperm in vaginal smears [23]. After mating, all pregnant rats were placed in individual cages to avoid gestational stress. As shown in Figure 1, thirty pregnant rats at E0.5 were randomly allocated to either a control diet group or a treatment group that received the diet supplemented with 20 mg/kg hArg (as-dry matter basis). Each group consisted of 15 biological replicates. The E13 was identified as the critical time point for the development of the rodent placenta. Therefore, the treatment group received hArg supplementation from E0.5 to E13. During the experiment, all rats had free access to water and a standard diet, the detailed composition of which is provided in Table S1.

### 2.2. Tissue Collection

For sample collection on E21, all pregnant rats were anesthetized with isoflurane. Blood samples were collected via cardiac puncture, subsequently subjected to centrifugation at 4 °C and 3000 rpm for 15 min to isolate the plasma, which was then cryopreserved at −80 °C. Following euthanasia through cervical dislocation, the abdomen and uterus were accessed via cesarean section to obtain placental tissue and labeled according to position in each uterine horn. To ensure consistency, placental samples from the proximal, middle, and distal regions of the left uterine horn were rapidly frozen in liquid nitrogen and stored at −80 °C for subsequent molecular analyses. Corresponding tissue samples from the right uterine horn were fixed in 4% paraformaldehyde for histological evaluation.

### 2.3. Pregnancy Outcome

The body weights of pregnant rats were weighed at E0.5 and E21, respectively. Feed intake was measured at 13:00 throughout the pregnancy. Following the cesarean section, the number of live litters delivered, live fetus weight, litter weight, placental weight, and total placental weight were all recorded.

### 2.4. Amino Acid Analysis

The hArg concentration in the placenta was quantified utilizing the hArg standard (H1007; Sigma-Aldrich, St. Louis, MO, USA) and a Waters H-Class Ultra Performance Liquid Chromatograph system (UPLC; Waters, Milford, MA, USA) equipped with an ACQUITY UPLC BEH C18 column. The system was equilibrated with a mixture comprising 86% 0.1 M sodium acetate and 14% chromatographic grade methanol. The samples were ultimately pretreated following the previously outlined method for hArg content analysis.

### 2.5. Assessment of Reproductive Hormones

Estradiol (E_2_) and progesterone (PROG) levels in rat plasma were assessed using a commercial enzyme-linked immunosorbent assay (ELISA) kit (mlbio, Shanghai, China) according to the manufacturer’s protocol. Briefly, plasma and standard dilutions were added to pre-coated plate wells. After adding the HRP-conjugated detection antigen, plates were incubated at 37 °C for 60 min. Following five thorough washes, a substrate solution (a mixture of solutions A and B) was introduced and allowed to incubate for 15 min at 37 °C in darkness before the reaction was stopped. Absorbance was read at 450 nm using a microplate reader (BioTeck, Winooski, VT, USA), and sample concentrations were calculated via four-parameter logistic curve fitting of standard curves.

### 2.6. Histologic Analysis of the Placenta

The placental histology was supported by Jijia Bio (Shenyang, China). The placenta was carefully removed and fixed in a 4% paraformaldehyde solution. The specimens were treated utilizing standard histological techniques, encompassing dehydration with ethanol and xylene, and were ultimately embedded in paraffin. To ensure a comprehensive and representative morphological analysis, a minimum of three sagittal sections (4 μm thickness) from the central region of each placenta were prepared and mounted on separate slides. Sections were deparaffinized by sequentially immersing them in xylene and progressively diluted ethanol solutions before staining with hematoxylin and eosin. Histological investigation was performed utilizing a panoramic slide scanner (Pannoramic DESK/MIDI/250/1000, 3DHISTECH, Budapest, Hungary). The entire placenta was imaged with CaseViewer (version 2.4, 3DHISTECH, Budapest, Hungary) at suitable magnification to measure the area of the placental LZ and junction zone (JZ). Furthermore, photographs of three randomly selected fields of interest under a high-magnification microscope were collected for each section, and the photographs were algorithmically trained and integrated into automated images by the AIpathwell^®^ software (version 2.1.1, Servicebio Technology Co., Ltd., Wuhan, China) to assess the positive area ratio of blood sinuses in the LZ.

### 2.7. Immunofluorescence Analysis of the Placenta

The placental immunofluorescence assays were supported by Jijia Bio (Shenyang, China). For a robust evaluation of microvascular density, a minimum of three sagittal sections (4 μm thickness) from the central region of each placenta were analyzed. Post-deparaffinization and rehydration, sections were immersed in citrate buffer for antigen retrieval. Thereafter, sections were blocked with 5% bovine serum albumin and treated with cluster of differentiation 31 (CD31; ab182981, Abcam, Cambridge, UK) at 4 °C overnight. Following washing in phosphate-buffered saline, sections were incubated with fluorescently tagged secondary antibodies (JJ0051, Jijia Bio, Shenyang, China) and counterstained with DAPI. All stained sections were scanned with a panoramic section scanner (Pannoramic DESK/MIDI/250/1000, 3DHISTECH, Budapest, Hungary). For quantitative analysis of CD31 integrated density, mean gray value, and microvascular density, six non-overlapping fields within the LZ were randomly selected and captured from each sections using Case Viewer (version 2.4, 3DHISTECH, Budapest, Hungary). Quantitative analysis was conducted using ImageJ (version 1.53a, National Institutes of Health, Bethesda, MD, USA). The results from all fields across all sections for all given placenta were averaged, and this average value was then used to represent that single biological replicate in the final statistical analysis.

### 2.8. Quantitative Polymerase Chain Reaction (qPCR)

Total RNA was extracted from the placenta utilizing Trizol (Takara, Kyoto, Japan). The quality of the RNA was evaluated by determining the absorbance ratio at 260 nm/280 nm, with ratios between 1.8 and 2.0 signifying satisfactory purity. Reverse transcription was conducted following the instructions of the One-Step gDNA Removal Reverse Transcription Kit (TransGen Biotech, Beijing, China). The reverse transcription parameters were set at 50 °C for 5 min, succeeded by 85 °C for 2 min. The generated cDNA was stored at −80 °C for future application. Gene sequences were obtained from GenBank. Primers were designed based on conserved regions and synthesized by Sangon Biotech Co., Ltd. (Shanghai, China). The quantitative assessment of gene expression was conducted utilizing the PerfectStart Green qPCR SuperMix (TransGen Biotech, Beijing, China). The cycle threshold (Ct) was determined utilizing the StepOne Plus Real-Time PCR System (Applied Biosystems, Waltham, MA, USA), and relative mRNA expression levels were calculated employing the 2^−ΔΔCt^ method, with β-actin serving as the internal reference gene for normalization. Detailed primer information is provided in Table S2.

### 2.9. Western Blotting

The placental tissue was homogenized in RIPA buffer (Thermo Fisher, Waltham, MA, USA), and the protein concentration of the supernatant was assessed using the Pierce™ BCA Protein Quantification Kit (Thermo Fisher, Waltham, MA, USA). The supernatant was mixed with the 5× SDS-PAGE loading buffer (Kangwei Century, Taizhou, China) and thereafter treated to heat denaturation for application in SDS-polyacrylamide gel electrophoresis (SDS-PAGE). Following the transfer of the protein from the gel to the PVDF membrane (Millipore, Billerica, MA, USA), it was subjected to blocking with 5% fetal bovine serum at room temperature for 2 h, subsequently treated with the primary antibody at 4 °C overnight. The utilized antibodies comprise vascular endothelial growth factor A (VEGFA; Abcam, Cambridge, UK), endothelial nitric oxide synthase (eNOS; Abclonal, Wuhan, China), phosphoinositide 3-kinase (PI3K 110; Affinity, San Francisco, CA, USA), AKT serine/threonine kinase (AKT; Abclonal, Wuhan, China), and phosphorylated AKT (pAkt S473; #AP1208, Abclonal, Wuhan, China). After a wash with phosphate-buffered saline containing 0.1% Tween 20, the membrane was incubated at room temperature with the biotinylated secondary antibody conjugated to HRP (Thermo Fisher, Waltham, MA, USA) for 1 h. The membrane was subsequently incubated in the ECL chemiluminescence reagent kit (NCM Bio, Shanghai, China) for protein band detection, and the images were visualized with the IS4000 chemical isotope imaging system (Kodak, Rochester, NY, USA). The signal intensity of the target protein was measured utilizing ImageJ software (version 1.53a, National Institutes of Health, Bethesda, MD, USA).

### 2.10. Statistical Analysis

To ensure the reliability of the findings, potential biases were minimized through random allocation of animals and blinded assessment during data collection and analysis. Possible contaminations in molecular analyses were controlled by using dedicated instruments for tissue processing, maintaining separate work areas, and including negative controls. Finally, data were presented as mean ± standard deviation (SD), and statistical analysis was performed using SPSS Statistics 26 software (version 26, IBM Corporation, Armonk, NY, USA). After doing the Shapiro–Wilk test and the Brown–Forsythe test to evaluate the normality and equal variance of the data, an independent samples t-test was utilized to compare the two groups. Differences were deemed highly significant for *p* < 0.01, significant for *p* < 0.05, and indicative of a trend for 0.05 ≤ *p* < 0.1. Data visualization graphs were generated using GraphPad Prism 10 (version 10.1.2, GraphPad Software, San Diego, CA, USA).

## 3. Results

### 3.1. Maternal hArg Supplementation Ameliorates Pregnancy Outcomes at E21

**Figure 2:**
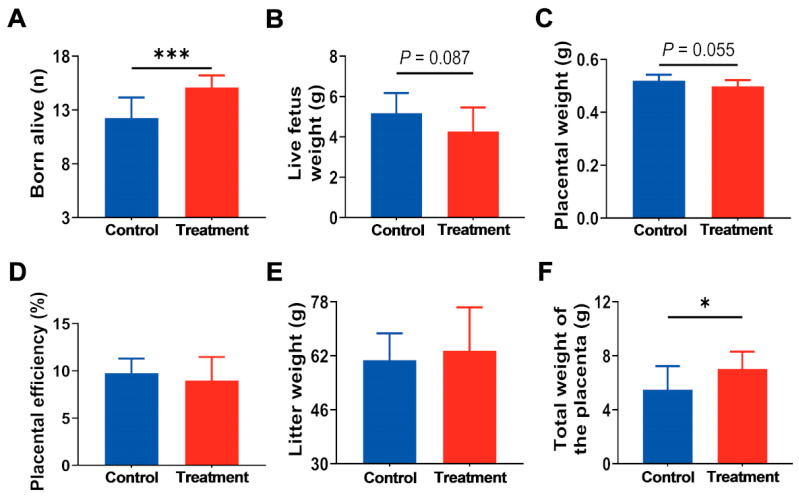
The pregnancy outcome of rats at embryonic day 21 (E21) was altered by hArg supplementation during the rapid development period of the placenta. (**A**) Total number of live fetuses. (**B**) The birth weight of a live fetus. (**C**) Placental weight. (**D**) Placental efficiency = fetal weight/placental weight. (**E**) Total weight of live fetuses. (**F**) The total weight of the placenta. The mean ± standard deviation (SD) is used to express the values. *n* = 15 biological replicates per group. * *p* < 0.05, *** *p* < 0.001.

Maternal rats were supplied with a 20 mg/kg hArg diet from E0.5 to E13, and pregnancy outcomes were assessed at E21. No significant variations in maternal feed intake and body weight were observed between the two groups (*p* > 0.05; Figure S1A–D). In comparison to the control group, the treatment group receiving 20 mg/kg of hArg during the rapid placental development period exhibited a significant increase in the number of live pups delivered (*p* < 0.05; Figure 2A). It is worth noting that the live fetus weight (4.27 ± 1.18 g vs. 5.18 ± 0.99 g; *p* = 0.087) and placenta weight (0.50 ± 0.02 g vs. 0.52 ± 0.02 g; *p* = 0.055) in the hArg treatment group were numerically lower than those in the control group, exhibiting a declining trend; however, these differences did not achieve statistical significance (Figure 2B,C). Furthermore, the fetoplacental weight ratio, an indicator of placental efficiency, did not differ significantly between the two groups (*p* > 0.05; Figure 2D). Importantly, the total weight of surviving fetuses and the total weight of the placenta in the hArg supplementation group increased by 4.63% (*p* > 0.05) and 27.64% (*p* < 0.05), respectively, in comparison to the control group (Figure 2E,F). The data suggest that hArg dietary supplementation during the rapid development period of the placenta enhances pregnancy outcomes, despite a tendency for decreased individual fetal and placental weights, while overall fetal-placental developmental efficiency is optimized.

### 3.2. Maternal hArg Supplementation Affected the Reproductive Endocrine System of Pregnant Rats

**Figure 3:**
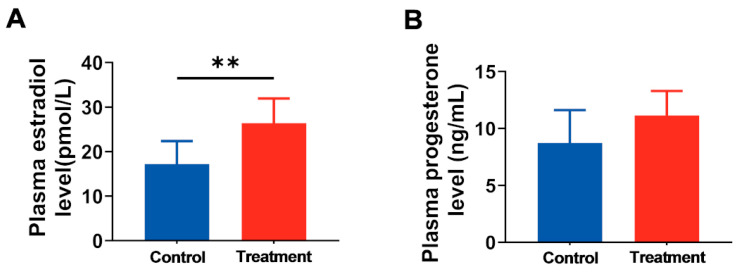
Administration of hArg during the rapid development period of the placenta affected maternal blood reproductive hormone levels. (**A**) The ELISA was employed to compare the plasma estradiol levels of the various groups. (**B**) Plasma progesterone levels. The mean ± standard deviation (SD) is used to express the values. *n* = 6 biological replicates per group. ** *p* < 0.01.

Sex steroids play a crucial role in regulating various physiological processes, including reproduction and development [24]. To investigate whether hArg affects hormone synthesis and secretion, we quantified the levels of E_2_ and PROG in maternal plasma. The research revealed that hArg supplementation during the rapid development period of the placenta significantly elevated maternal plasma E_2_ levels compared to the control group (26.42 ± 5.50 pmol/L vs. 17.23 ± 5.14 pmol/L; *p* < 0.05; Figure 3A). Moreover, plasma PROG levels increased in the hArg-treated group (11.15 ± 2.15 ng/mL vs. 8.74 ± 2.88 ng/mL), but no statistically significant difference was observed between the two groups (*p* > 0.05; Figure 3B). The findings indicate that hArg supplementation promotes the synthesis and secretion of reproductive hormones, suggesting a potential role for hArg in regulating pregnancy.

### 3.3. Supplementation of Maternal hArg Promotes hArg Transport and Metabolism in the Placenta

**Figure 4:**
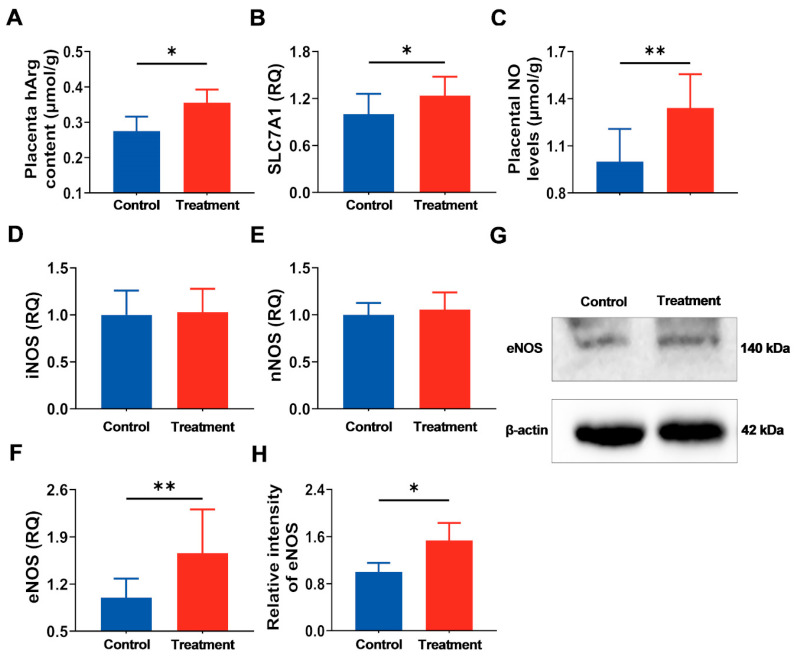
Application of hArg during the placental development period augmented the transport and metabolism of hArg within the placenta. (**A**) The hArg content in the placenta. (**B**) Gene expression of *solute carrier family 7 member 1 (SLC7A1)* within the placenta. (**C**) Nitric oxide (NO) levels in the placenta. (**D**–**F**) Gene expression of *inducible nitric oxide synthase (iNOS)*, *neuronal NOS* *(nNOS)*, and *endothelial* NOS (*eNOS*). (**G**) Western blot analysis of eNOS protein. (**H**) eNOS protein levels. Protein levels were standardized utilizing β-actin as a reference and expressed relative to the control group. Values are presented as mean ± standard deviation (SD). *n* = 3–6 biological replicates per group. * *p* < 0.05, ** *p* < 0.01.

The hArg content of the placenta was measured to determine if exogenous hArg supplementation could effectively traverse the placental barrier and accumulate in the placenta. The treatment group exhibited a statistically significant increase in hArg content compared to the control group (*p* < 0.05; Figure 4A). The mRNA expression levels of the hArg transporter *solute carrier family 7 member 1* (*SLC7A1*) in the placentas of both groups were consistent with hArg content, with upregulation in the treatment group (*p* < 0.05; Figure 4B). These findings indicate effective transport of hArg in the placenta.

It has been proven that hArg can produce NO by serving as a substrate for NOS [11,25]; hence, further research was undertaken to examine changes in NO levels and NOS expression in the placenta. Results showed that the hArg-treated group exhibited elevated NO levels in the placenta relative to the control group (*p* < 0.05; Figure 4C), implying that NOS was activated in the placenta. Analysis of NOS mRNA expression revealed that, compared to the control group, hArg treatment during the placental rapidly developing period did not yield statistically significant differences in *inducible NOS* (*iNOS*) and *neuronal NOS* (*nNOS*) mRNA expression (*p* > 0.05), yet it significantly elevated eNOS mRNA and protein expression levels (*p* < 0.05; Figure 4D–H). These results indicate that hArg enhances NO generation in the placenta predominantly by eNOS, rather than iNOS and nNOS.

### 3.4. Maternal hArg Supplementation Enhanced Placental Development at E21

**Figure 5:**
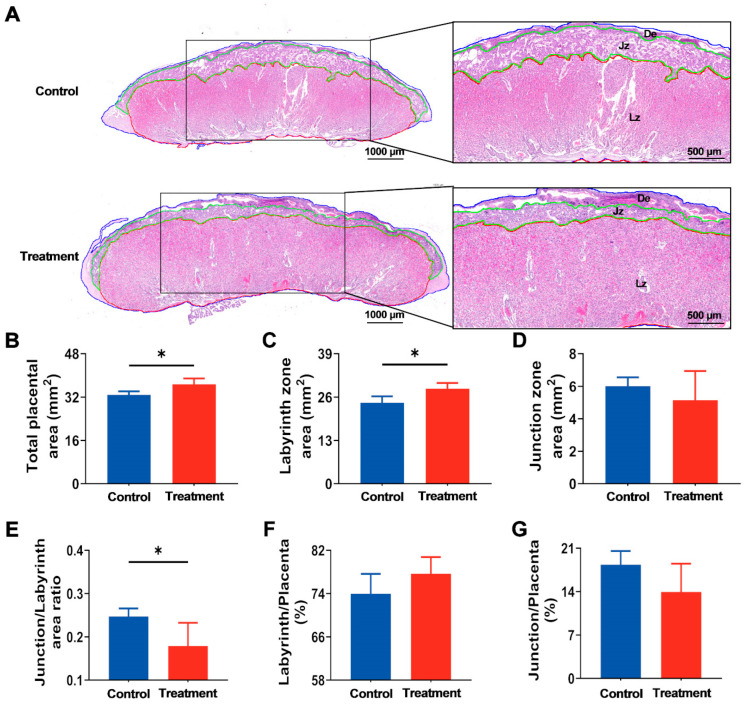
Application of hArg during the placental rapid developmental period causes adaptive remodeling of placental architecture. (**A**) Representative images of hematoxylin-eosin staining in the central region of the placenta within the sagittal section, with the second column magnifying the boxed regions in the first column; scale bars, 1000 µm (first column) and 500 µm (second column); the red lines indicate the labyrinth zone (LZ), the green lines indicate the junction zone (JZ), and decidual layer (DE) represents the decidua. (**B**) Total placental area. (**C**) LZ area. (**D**) JZ area. (**E**) JZ/LZ area ratio. (**F**) The percentage of area of LZ/whole placenta. (**G**) The percentage of area of JZ/whole placenta. Data are presented as mean ± standard deviation (SD). *n* = 4 biological replicates per group. * *p* < 0.05.

Subsequently, to assess the effect of hArg on placental development, the morphology of the placenta was analyzed at E21. As shown in Figure 5A, histological examination of placental demonstrated significant structural alterations in the treated group. Specifically, the total area of the placenta in the treated group was significantly greater than that in the control group (*p* < 0.05; Figure 5B). The placental LZ establishes a barrier for maternal–fetal exchange [26]. A specific evaluation of LZ and JZ showed that, relative to the control group, hArg treatment during the rapid development period of the placenta significantly increased the LZ area by approximately 17.24% (*p* < 0.05), but the JZ area remained unchanged (Figure 5C,D). Additionally, the decrease in the JZ/LZ ratio in the hArg-treated group (*p* < 0.05; Figure 5E), further evidencing the notable enhancement in placental development induced by hArg. It is worth noting that no significant difference was observed between the two groups for the ratio of LZ and JZ to the total placental area (*p* > 0.05; Figure 5F,G). The aforementioned data demonstrate that hArg particularly enlarges the LZ area of the placenta, enhances its functional structure, and facilitates placental growth.

### 3.5. Maternal hArg Supplementation Facilitates Placental Angiogenesis

**Figure 6:**
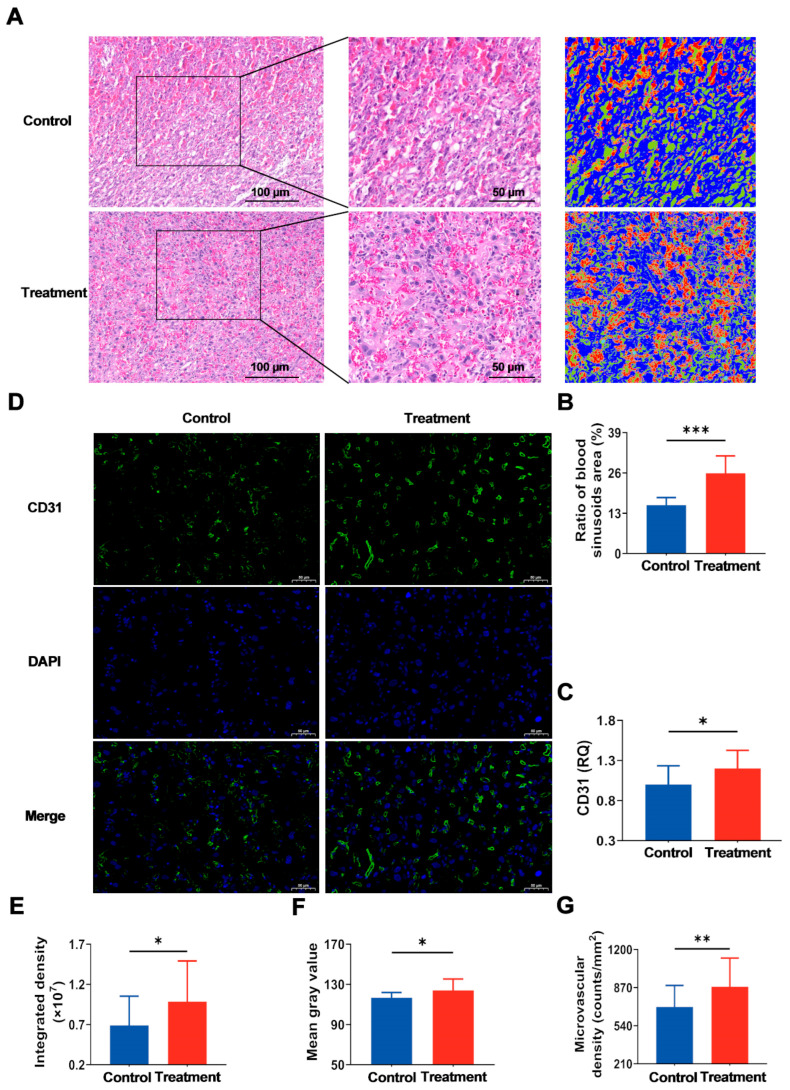
Application of hArg during the placental rapid development period enhances angiogenesis in the labyrinth zone (LZ). (**A**) Examples of blood sinusoids in the LZ stained with hematoxylin and eosin; the second column presents a magnification of the boxed region in the first column; the third column displays an analytical image produced by the Aipathwell software following positive assessment and grading of the second column image; scale bar, 100 µm (first column) and 50 µm (second and third columns); red-filled regions in the analytical image denote blood sinusoids. (**B**) Ratio of positive blood sinusoids. (**C**) Gene expression levels of cluster of differentiation 31 (CD31) in placenta. (**D**) Exemplary images of placental CD31 immunofluorescence staining, showing green (CD31), blue (nuclei, DAPI), and merged images; scale bar, 50 µm. (**E**) Integrated density of CD31. (**F**) Mean gray value. (**G**) Microvascular density. Data are expressed as mean ± standard deviation (SD). *n* = 4 biological replicates per group. * *p* < 0.05, ** *p* < 0.01, *** *p* < 0.001.

To initially determine the impact of hArg on the placental vascular network, the area of blood sinusoids in the LZ was analyzed using hematoxylin and eosin staining of the placenta (Figure 6A). The results showed that the treatment group had a markedly elevated positive rate for the blood sinusoids region in the LZ relative to the control group (*p* < 0.05; Figure 6B). CD31, a commonly utilized marker for evaluating in vivo angiogenesis [27], exhibited a significant upregulation of its mRNA levels in the hArg-treated group (*p* < 0.05; Figure 6C). Consistent with this, immunofluorescence analysis of CD31 in the placenta revealed that hArg supplementation significantly improved the integrated density and mean gray value of CD31 in the placental LZ compared to the control group, by approximately 42.69% and 6.29%, respectively (*p* < 0.05; Figure 6D–F). Additionally, the microvascular density in the treatment group was approximately 24.94% more than in the control group (*p* < 0.05; Figure 6G). These findings demonstrate that hArg improves vascularization in the placental LZ, thereby optimizing the structure of the placental vascular network.

### 3.6. The hArg Activates the PI3K-AKT Pathway, Optimizing the Environment for Angiogenesis

**Figure 7:**
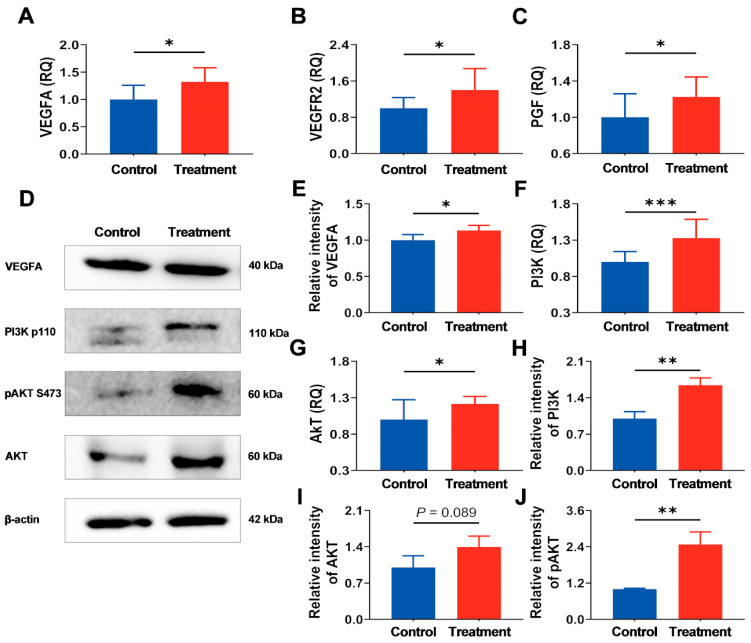
Administration of hArg during the placental rapid development period stimulated the PI3K-AKT pathway and increased the expression of angiogenesis-related proteins. The mRNA levels of *VEGFA* (**A**), *VEGFR2* (**B**), and *PGF* (**C**) in the placenta were analyzed by RT-qPCR. (**D**) Western blot analysis of VEGFA, PI3K, AKT, and pAKT. (**E**) Protein levels of VEGFA. Genes expression levels of the *PI3K* (**F**) and *AKT* (**G**). Pathway PI3K (**H**), AKT (**I**), and pAKT (**J**) protein levels. All gene and protein expression levels were normalized against β-actin as a reference and expressed relative to the control. Data are expressed as mean ± standard deviation (SD). *n* = 4 biological replicates for each group. * *p* < 0.05, ** *p* < 0.01, *** *p* < 0.001.

Figure 7A–C depict the mRNA expression data of factors associated with placental angiogenesis. VEGFA, *VEGF receptor 2* (*VEGFR2*), and *placental growth factor* (*PGF*) exhibited significant rises in the treatment group compared with the control group, with increases of 32.28%, 40.02%, and 22.52%, respectively (*p* < 0.05). Furthermore, hArg supplementation upregulates VEGFA protein expression (*p* < 0.05; Figure 7D,E), in keeping with its mRNA expression. These findings suggest that hArg enhances the expression of placental angiogenesis-related factors, fostering an environment conducive to angiogenesis.

Previous study proved that the PI3K-AKT pathway participates in the VEGF cascade and regulates placental angiogenesis [2]. Consequently, we conducted a more detailed analysis of the expression of proteins associated with the PI3K-AKT pathway in the placenta. As illustrated in Figure 7F,G, supplementation with hArg markedly elevated the mRNA expression of *PI3K* and *AKT* relative to the control group (*p* < 0.05). Simultaneously, the protein expressions of PI3K and pAKT in the treatment group were significantly higher than in the control group (*p* < 0.05), with AKT protein expression showing an upregulation (*p* = 0.089; Figure 7D,H–J). The results confirm that hArg supplementation activates the PI3K-AKT pathway in the placenta, forming a positive regulatory network that promotes placental angiogenesis.

## 4. Discussion

In this study, we observed that hArg supplementation during rapid placental growth ultimately altered pregnancy outcomes, placental morphology and transport, and placental angiogenesis in pregnant rats. Experimental data indicated that hArg treatment significantly increased the number of viable fetuses at term delivery while elevating maternal steroid hormone levels without significantly impacting maternal food intake or body weight throughout pregnancy. In vivo experiments demonstrated that hArg improves the placental nutrient transport environment and vascularization to modulate pregnancy outcomes. Notably, among the three NOS subtypes, eNOS is the principal enzyme facilitating hArg metabolism to produce NO, thereby regulating placental angiogenesis. Mechanistically, the augmentation of placental vascular endothelial function is predominantly ascribed to hArg’s promotion of phosphorylation in the PI3K-AKT pathway. These findings support hArg as a potential nutritional intervention strategy that enhances placental vascular development and ameliorates pregnancy outcomes through the activation of the PI3K/AKT signaling pathway.

The placenta serves as a central site for maternal–fetal exchange, executing essential functions necessary for fetal health and growth. Numerous studies reveal that anomalies in placental development are associated with preterm birth, low birth weight, preeclampsia, and decreased neonatal survival rates. These anomalies may also induce various chronic diseases from infancy to adulthood and may produce transgenerational health effects [28]. In numerous placental dysfunction models, increased NO production has been demonstrated to markedly improve placental perfusion and neonatal pregnancy outcomes [29,30,31]. Notably, Khalil AA et al. [32] found that in patients with early-onset preeclampsia, marked by impaired endothelial function, blood hArg levels were significantly reduced during early pregnancy (weeks 11–13). This reduction in hArg, as an endogenous substrate for NOS, could alter NO metabolism and production during early pregnancy, resulting in diminished NO bioavailability. Conversely, previous studies investigating the fluctuations in maternal blood hArg levels throughout normal pregnancy revealed significantly higher hArg concentrations during pregnancy relative to non-pregnant states, indicating that this amino acid may play a role in endothelial function [20,33]. Although these findings enhance our comprehension of the physiological and clinical importance of hArg during pregnancy, its potential regulatory function in healthy pregnancy and placental development remains unclear. The present study initially demonstrates that hArg supplementation at the administered dose produced no adverse effects on maternal physiological parameters. A groundbreaking human study has further confirmed that hArg supplementation is safe and well-tolerated [34], a conclusion substantiated by multiple investigations utilizing supraphysiological hArg concentrations [11,16,17,18,19]. As an endogenous amino acid, hArg further supports its biological compatibility, establishing a foundation for its potential as a promising candidate for clinical translation. More importantly, administration of 20 mg/kg hArg during the rapid phase of placental growth resulted in improvements in pregnancy outcomes, particularly a significant increase in the number of viable fetuses and total placental weight. Consistent with findings from Greene et al. [35], the rise in litter size was accompanied by reduced individual birth weight. A declining trend in individual liveborn fetal weight and single placental weight was also observed in our study; however, notably, the fetoplacental weight ratio (placental efficiency) remained stable across groups. These findings indicate that hArg supplementation enhances overall maternal reproductive performance while maintaining the functional integrity of individual placental units, highlighting its potential to beneficially modulate the pregnancy process.

As pregnancy progresses, E_2_ and PROG, as essential steroid hormones, are recognized as pivotal regulators of normal placental development, influencing vascular endothelial function and placental perfusion [36,37,38]. Studies indicate that maternal amino acids and reproductive hormones perform intricate bidirectional regulatory interactions during gestation. For example, regarding Arg, Spencer et al. [39] showed that plasma E_2_ and PROG in pregnant sows augment Arg transport in the placenta, hence enhancing its bioavailability. Correspondingly, in vitro supplementation of Arg showed a quadratic trend in the elevation of PROG in swine trophoblast cells [40]. In vivo experiments conducted by Gao et al. [41] have shown that dietary supplementation with Arg increased maternal plasma E_2_ levels. Similarly, in this study, we noted higher levels of E_2_ and PROG in maternal plasma in the hArg-treated group, though the difference in PROG levels between the two groups was not statistically significant. Subsequently, we conducted a further analysis of hArg content in the placenta and discovered that hArg supplementation increased hArg levels in placental tissue. As previously described, cationic amino acid transporters in the CAT family, namely SLC7, mediate the cellular uptake of hArg, with SLC7A1 acting as the primary transporter [19,42]. This study discovered that maternal hArg supplementation upregulates SLC7A1 mRNA expression in the placenta, consistent with elevated placental hArg levels. These results collectively indicate that dietary hArg supplementation promotes the secretion of essential steroid hormones, upregulates transporter expression, and synergistically enhances placental hArg uptake.

NOS is expressed in the placenta [43]. Numerous findings indicate that hArg, serving as an alternate substrate for NOS, can directly enhance the synthesis of protective NO [11,19]. Furthermore, NO has been found to be a regulator of placental blood flow perfusion, thereby facilitating the delivery of oxygen and nutrients [22]. Numerous studies have demonstrated that supplementation with NO precursors, such as Arg, can significantly improve clinical outcomes in high-risk pregnancies, highlighting the clinical significance of enhancing this pathway [30,44]. Significantly, whereas NO functions as the principal endothelium-derived relaxing factor exerting strong vasodilatory effects on systemic resistance vessels, this impact is primarily mediated by locally produced NO [22,45]. To more precisely assess whether hArg is bioavailable in the placenta and thereby exerts its potential regulatory effects, we quantified NO production and NOS expression in the placenta. The current study indicated that hArg increased NO production in the placenta, consistent with previous reports [12,21]. These results suggest that hArg raises NO availability and activates NOS in the placenta. In mammals, NOS is predominantly categorized into three subtypes, including iNOS, nNOS, and eNOS [46]. Our study found that hArg supplementation significantly elevated eNOS mRNA levels in the placentas of pregnant rats, without impacting the mRNA expression levels of iNOS and nNOS. Moreover, the protein expression of eNOS exhibited consistent results with its mRNA, showing a significant improvement in the hArg-treated group. This corresponds with eNOS’s status as a key constitutive enzyme essential for sustaining vascular homeostasis in endothelial cells [47]. In contrast, Nitz et al. [19] reported that supplementation with [^15^N_4_^13^C_7_]-hArg and [^15^N_2_]-Arg in atherosclerotic mouse models did not result in significant variations in urine [^15^N]-nitrate, suggesting that neither hArg nor Arg affected NO production regulated by NOS in vivo. This discrepancy may stem from the intrinsic discrepancies between the physiological model of pregnant rats and the pathological model of atherosclerotic mice. Additionally, another important factor is that the effects of NO are localized. The present study directly assessed NO produced by hArg metabolism in the placenta. However, the detection of NO metabolites in urine reflects NO production by hArg throughout the systemic organism. NO metabolites undergoing dilution in the systemic circulation may have led to levels falling below the detection threshold, thereby not excluding the potential for hArg to locally produce NO in particular tissues and have biological effects. Consequently, discrepancies in research models (physiological/pathological) and detection methodologies (local/systemic) may explain the divergent outcomes of hArg on NO production. Overall, our findings suggest that dietary supplementation with hArg specifically upregulates placental eNOS expression and promotes local NO production, ultimately improving placental function.

Well-regulated angiogenesis is crucial for various physiological processes. Prior research indicated that placental vascular development constitutes a critical component of normal placental function [48]. The efficiency of nutrient transport from the pregnant mother to the developing fetus predominantly relies on the maturation of the placental vascular network, which is frequently utilized to evaluate placental efficiency [1,49]. First, we analyzed placental morphology using HE staining and discovered that hArg supplementation altered placental morphology, but there was no significant difference in placental weight between the two groups. Zhang et al. [1] found analogous results, demonstrating that while dietary folate supplementation did not affect placental weight, it enhanced placental morphology. This finding aligns with the results reported by Bao et al. [50]. The rodent placenta comprises three different regions: the LZ, JZ, and decidual layer [51]. In the mature placenta, the LZ is the tissue layer nearest to the fetus and functions as the primary location for maternal–fetal nutrition exchange [1,52]. Specifically, this study shows that the total placental area significantly increased in the dietary hArg supplementation group, with this augmentation predominantly due to an absolute increase in the LZ rather than the JZ. Nonetheless, hArg treatment did not influence the ratios of LZ and JZ to the total placental area, likely because hArg facilitates coordinated general growth in the placenta. In comparison to other placental regions, the LZ possesses the most abundant vascular network, and the placenta’s better transport of nutrients is mostly reliant on its high degree of vascularization [53]. As previously noted, alterations in the LZ reflect changes in the placental vascular structure [54]. This work demonstrated that hArg treatment augmented the area of blood sinuses in the LZ and enhanced the expression of the in vivo angiogenesis marker CD31 in the placenta. Furthermore, placental immunofluorescent histochemical staining results demonstrated that hArg supplementation elevated the integrated density values and mean gray value of placental CD31, generally signifying an increase in placental vascular number. Likewise, the rise in microvascular density further substantiates the conclusion that hArg facilitates placental angiogenesis. Consequently, hArg facilitates the placental architecture’s coordinated remodeling and angiogenesis, implying enhanced placental nutrient exchange capacity to compensate for increased inter-fetal nutrient limitations and support fetal growth.

The complex process of placental angiogenesis is regulated by angiogenesis-related factors, essential for the successful establishment of early placental vascularization [1,55]. Among these, VEGFA occupies a central position in angiogenesis. The principal signaling receptor is VEGFR2, which, by specific binding, induces endothelial cell proliferation and migration and thus promotes angiogenesis [2,55,56]. Moreover, the co-expression of PGF and VEGFA inside the same cell results in the formation of heterodimers that synergistically enhance endothelial cell mitosis upon receptor binding [49,57]. Evidence suggests that VEGF relies on the PI3K/AKT pathway and can enhance eNOS expression, establishing a PI3K/AKT/VEGF/eNOS axis that increases NO production. This phenomenon relates to the established function of PI3K-AKT in affecting endothelial cells [58,59]. The evidence we provide indicates that hArg enhances the expression of VEGFA, VEGFR2, and PGF, while concurrently upregulating proteins in the PI3K-AKT pathway. Consistent with our findings, numerous studies have emphasized the pivotal regulatory role of the PI3K/Akt/VEGF signaling pathway in angiogenesis [4,60]. In conclusion, these findings suggest that hArg activates the PI3K/Akt/VEGF pathway in placental tissue, mutually corroborated by the elevation of eNOS expression and increased NO production.

This study demonstrates important strengths through its comprehensive approach that combined physiological, histological and molecular analyses. The research design enabled precise hArg supplementation during the critical window of placental development while successfully elucidating the complete signaling pathway from hArg transport through PI3K/AKT activation to functional angiogenesis. Nevertheless, this study includes some limitations. At first, while we observed the upregulation of angiogenesis-related factors in the placenta, significant proangiogenic phenomena, and the activation of the PI3K/AKT pathway, we did not employ a rat model with a placenta-specific blockade of this pathway to further confirm the necessity of PI3K/AKT in hArg-mediated angiogenesis. However, previous studies support the scientific feasibility that PI3K/AKT/VEGF promotes angiogenesis through the augmentation of NO production. Furthermore, we performed comprehensive analyses of critical downstream genes and proteins, which improved the signaling cascade. Secondly, while the positive effects of hArg on placental angiogenesis and pregnancy outcomes are evident, its potential to enhance placental function in pathological circumstances requires additional research. Additionally, this study was limited to evaluating outcomes at term and did not investigate the potential long-term effects of hArg supplementation on postnatal offspring health and development. Ultimately, owing to interspecies differences, the positive effects of hArg on placental angiogenesis and pregnancy outcomes cannot be directly extrapolated to human populations or other animal species. In future investigations, we intend to apply a relatively easy-to-operate in vitro vascular endothelial cell model, utilizing specific pathway protein inhibitors to preliminarily validate the molecular mechanism by which hArg regulates angiogenesis. Furthermore, given the potential applicability of hArg’s beneficial effects on placental angiogenesis and pregnancy outcomes to physiologically stressed pregnancies, we will further assess its therapeutic potential in animal models of placental dysfunction (such as COVID-19 pandemic [61], intrauterine growth restriction [62], and preeclampsia [63], etc., which are associated with adverse perinatal outcomes) to clarify its utility in various clinical conditions. We will also specifically explore the potential long-term impacts of prenatal hArg exposure on postnatal growth and metabolic health of the offspring. Simultaneously, for human populations, we may collect blood and placental samples from patients with placental dysfunction problems to further elucidate hArg’s physiological significance in human pregnancy and its prospective clinical applications.

## 5. Conclusions

In summary, this study confirms that the regulatory effect of hArg on pregnancy outcomes can be attributed to enhanced placental amino acid uptake, which activates PI3K/AKT pathway proteins and increases the transcription of angiogenesis-related factors. This further promotes placental eNOS expression and local NO production, ultimately optimizing the vascular network structure and nutrient transport capacity of the LZ. These findings provide evidence for the physiological mechanism by which hArg improves placental function in healthy pregnancies and establish a theoretical foundation for its potential application in nutritional intervention strategies.

## Data Availability

The data presented in this study are available on request from the corresponding authors.

## Supplementary Materials

The following supporting information can be downloaded at: https://www.mdpi.com/article/10.3390/nu17223563/s1, Table S1: Ingredients and chemical formulation of experimental diets for rats; Table S2: Primer sequences for the polymerase chain reaction; Figure S1: Administration of hArg did not affect maternal feed intake, body weight, or weight gain during gestation. (A) Feed intake. (B) Maternal body weight at E0.5 and E21. (C) Weight gain during gestation. The gestational weight gain = maternal body weight at E21 − maternal body weight at E0.5. (D) Weight gain during gestation relative to body weight. Relative gestation weight gain = gestation weight gain/maternal body weight at E21 × 100%. The mean ± standard deviation (SD) is used to express the values. *n* = 15 biological replicates per group. * *p* < 0.05, ** *p* < 0.01, *** *p* < 0.001.

## Abbreviations

The following abbreviations are used in this manuscript:

Embryonic day 9.5	E9.5
LZ	Labyrinthine zone
hArg	Homoarginine
Arg	Arginine
NO	Nitric oxide
E_2_	Estradiol
PROG	Progesterone
JZ	Junction zone
CD31	Cluster of differentiation 31
VEGFA	Vascular endothelial growth factor A
eNOS	Endothelial nitric oxide synthase
PI3K	Phosphoinositide 3-kinase
AKT	AKT serine/threonine kinase
pAkt	Phosphorylated AKT
SLC7A1	Solute carrier family 7 member 1
iNOS	Inducible nitric oxide synthase
nNOS	Neuronal nitric oxide synthase
VEGFR2	Vascular endothelial growth factor receptor 2
PGF	Placental growth factor
