# Supplementary material for: Supplementation with hArg During the Rapid Growth of the Placenta Modulates Final Placental Angiogenesis and Pregnancy Outcomes
Source: Nutrients. 2025 Nov 14;17(22):3563. doi: 10.3390/nu17223563 (PMC12655641; doi:10.3390/nu17223563)
Supplement: Supplementary file 1 [file nutrients-17-03563-s001.zip › nutrients-3908182-supplementary.pdf]

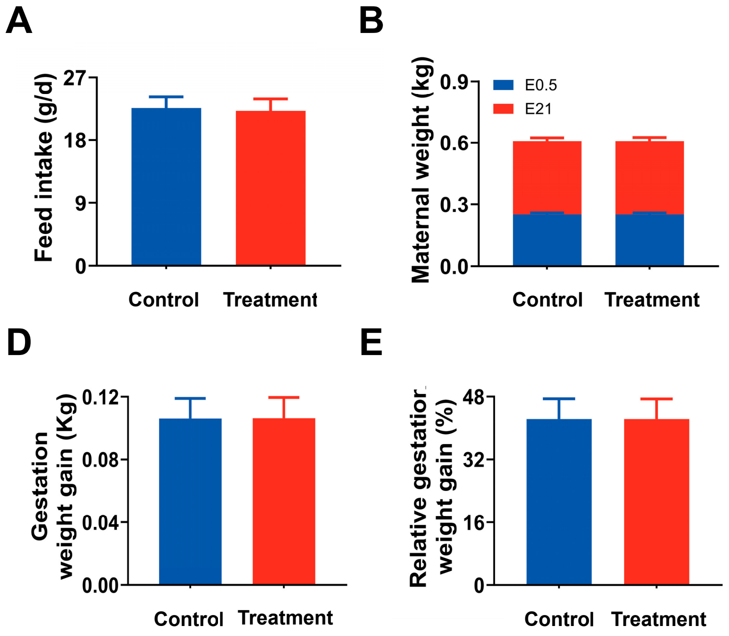

Figure S1. Administration of hArg did not affect maternal feed intake, body weight, or weight gain during gestation. (A) Feed intake. (B) Maternal body weight at E0.5 and E21. (C) Weight gain during gestation. The gestational weight gain = maternal body weight at E21 - maternal body weight at E0.5. (D) Weight gain during gestation relative to body weight. Relative gestation weight gain = gestation weight gain / maternal body weight at E21 × 100%. The mean ± standard deviation (SD) is used to express the values. n = 15 biological replicates per group. * $P < 0.05$, ** $P < 0.01$, *** $P < 0.001$.

Table S1. Ingredients and chemical formulation of experimental diets for rats.

Ingredient (as-fed basis), g/kg		Nutrition content	
Corn	48.75	NE, kcal/kg	3770.00
Flour	8.50	CP, g/kg	196.00
Soybean meal	15.50	EE, g/kg	65.00
Stone powder	2.00	Trp, g/kg	2.11
Fish meal	2.50	Thr, g/kg	6.72
Wheat bran	17.00	Met, g/kg	4.61
Calcium bicarbonate	0.50	Lys, g/kg	13.00
Sodium chloride	0.75	AP, g/kg	0.30
Vitamin and mineral premix[a]	4.50	Ca, g/kg	0.54
Total	100.00		

NE: Net energy; CP: Crude protein; EE: Ether Extract; Trp: Tryptophan; Thr: Threonine; Met: Methionine; Lys: Lysine; AP: Available phosphorus; Ca: Calcium.

[a] Supplied the following per kilogram complete diet, Vitamin A, 7000 IU; Vitamin B, 66 mg; Vitamin B1, 8 mg; Vitamin B2, 10 mg; Vitamin B12, 0.02 mg; Vitamin K, 3 mg; Vitamin D, 800 IU; Vitamin E, 60 IU; K, 5 g; Fe, 100 mg; I, 0.5 mg; Na, 2 g; Zn, 30 mg; Mg, 2 g; Mn, 75 mg. Mn, 75 mg.

Table S2. Primer sequences for the polymerase chain reaction.

Genes	Primer sequences (5′-3′)	Accession number
SLC7A1	Forward: CCCGCTGCCTCAACACCTATG<br>Reverse: CGATCAGGAAGGAGATGACGATGG	NM_001399982.1
iNOS	Forward: TCTTGGAGCGAGTTGTGGATTGTTC<br>Reverse: AGTGATGTCCAGGAAGTAGGTGAGG	NM_012953.2
nNOS	Forward: AATGGTGGAGGTGCTGGAGGAG<br>Reverse: GTCTGGAGAGGAGCTGATGGAGTAG	NM_012613.3
eNOS	Forward: GCCACCTGATCCTAACTTGCCTTG<br>Reverse: TCGTGTAATCGGTCTTGCCAGAATC	NM_021838.2
CD31	Forward: AGCCGACATTGTGACCAGTCTC<br>Reverse: TCAAGGCGGCAATGACCACTC	NM_031591.2
PGF	Forward: TCTCAGGATGTGCTCTGCGAATG<br>Reverse: CTCAGTCTGTGGGGTTTTGCTTTG	NM_053595.2
VEGFA	Forward: CCGTCCTGTGTGCCCCTAATG<br>Reverse: ATCTCTCCTATGTGCTGGCTTTGG	NM_001110333.2
VEGFR2	Forward: CAATTCCCGTCCTCAAAGCATCAG<br>Reverse: TTCTCCTTGGTCACTCTTGGTCAC	NM_013062.2
PI3K	Forward: GCCGATCCTACAGTCCTATCCAATG<br>Reverse: GCAGAAGGCACAGGTCCAGAG	NM_001371300.24
AKT	Forward: AGAAGGAGGTCATCGTTGCCAAG<br>Reverse: GCGGTCGTGGGTCTGGAATG	NM_033230.3
β-actin	Forward: TAGGCACCAGGGTGTGATGGTG<br>Reverse: ATCTTCTCCATGTCGTCCCAG	NM_031144.3

SLC7A1: Solute carrier family 7 member 1; iNOS: Inducible nitric oxide synthase; nNOS: Neuronal nitric oxide synthase; eNOS: Endothelial nitric oxide synthase; CD31: Cluster of Differentiation 31; PGF: Placental Growth Factor; VEGFA: Vascular endothelial growth factor A; VEGFR2: Vascular endothelial growth factor receptor 2; PI3K: phosphatidylinositol 3-kinase; AKT: AKT Serine/Threonine Kinase; β-actin: Beta-actin.
